# Germ-line variants identified by next generation sequencing in a panel of estrogen and cancer associated genes correlate with poor clinical outcome in Lynch syndrome patients

**DOI:** 10.18632/oncotarget.5694

**Published:** 2015-10-22

**Authors:** Balazs Jóri, Rick Kamps, Sofia Xanthoulea, Bert Delvoux, Marinus J. Blok, Koen K. Van de Vijver, Bart de Koning, Felicia Trups Oei, Carli M. Tops, Ernst J. M. Speel, Roy F. Kruitwagen, Encarna B. Gomez-Garcia, Andrea Romano

**Affiliations:** ^1^ Department of Gynecology and Obstetrics, GROW – School for Oncology & Developmental Biology, Maastricht University Medical Centre, The Netherlands; ^2^ Department of Clinical Genetics, GROW – School for Oncology & Developmental Biology, Maastricht University Medical Centre, The Netherlands; ^3^ Department of Pathology, GROW – School for Oncology & Developmental Biology, Maastricht University Medical Centre, The Netherlands; ^4^ Department of Clinical Genetics, Genomics & Bioinformatics, CARIM – School for Cardiovascular Diseases, Maastricht University Medical Centre, The Netherlands; ^5^ Department of Clinical Genetics, Leiden University Medical Centre, The Netherlands; ^6^ Current address: Divisions of Diagnostic Oncology & Molecular Pathology, Netherlands Cancer Institute-Antoni van Leeuwenhoek, Amsterdam, The Netherlands

**Keywords:** Lynch syndrome, endometrial cancer, genetic risk modifier, next generation sequencing, estrogens

## Abstract

**Background:**

The risk to develop colorectal and endometrial cancers among subjects testing positive for a pathogenic Lynch syndrome mutation varies, making the risk prediction difficult. Genetic risk modifiers alter the risk conferred by inherited Lynch syndrome mutations, and their identification can improve genetic counseling. We aimed at identifying rare genetic modifiers of the risk of Lynch syndrome endometrial cancer.

**Methods:**

A family based approach was used to assess the presence of genetic risk modifiers among 35 Lynch syndrome mutation carriers having either a poor clinical phenotype (early age of endometrial cancer diagnosis or multiple cancers) or a neutral clinical phenotype. Putative genetic risk modifiers were identified by Next Generation Sequencing among a panel of 154 genes involved in endometrial physiology and carcinogenesis.

**Results:**

A simple pipeline, based on an allele frequency lower than 0.001 and on predicted non-conservative amino-acid substitutions returned 54 variants that were considered putative risk modifiers. The presence of two or more risk modifying variants in women carrying a pathogenic Lynch syndrome mutation was associated with a poor clinical phenotype.

**Conclusion:**

A gene-panel is proposed that comprehends genes that can carry variants with putative modifying effects on the risk of Lynch syndrome endometrial cancer. Validation in further studies is warranted before considering the possible use of this tool in genetic counseling.

## INTRODUCTION

Lynch Syndrome is caused by mutations in one out of four mismatch repair (MMR) genes - *MLH1*, *MSH2*, *MSH6* and *PMS2* - and results in a 25% to 75% lifetime risk of colorectal cancer and 60% risk of endometrial cancer in women [1].

Genetic testing helps estimating the individual risk, plan appropriate care, screening and prophylactic treatments. Nevertheless, the risk to develop cancer conferred by a MMR mutation is modified by both the environment and genetic risk modifiers. The identification of such genetic risk modifiers can improve risk prediction and genetic counseling, through the individualization of surveillance programs and the evaluation of benefits *versus* burdens associated with prophylactic strategies [1, 2].

Searching for genetic risk modifiers is a challenge due to their expected small effect-size and their non-pathogenicity in the absence of a pathogenic mutation [2–4]. Genome-wide-association studies have identified loci and single nucleotide polymorphisms (SNPs) with risk modifying effects [5–8]. More recently, Next Generation Sequencing (NGS) of the whole genome or combined with gene-panels is emerging in genetic diagnostic to detect germ-line variants predisposing to cancer [4, 9–11].

Here, NGS combined with a 154 gene-panel was used to identify rare variants (minor allele frequency, MAF, <0.001) acting as gene-modifiers of Lynch syndrome MMR mutations. Unlike cancer somatic mutations, which map exclusively on tumor suppressors and oncogenes, the carcinogenic effect of germ-line variants can be indirect [2, 4, 11], hence they can map within but also outside the genes classically associated with cancer. A defect in the genome stability conferred by a Lynch syndrome MMR mutation can be aggravated by disturbed tissue homeostasis. Therefore, as proof of principle for Lynch syndrome related endometrial cancer, a panel that included genes controlling the endometrial physiology and homeostasis (i.e. hormone signaling) beside those associated with cancer was complied. A family based approach was used: the presence of candidate genetic risk modifiers was evaluated among 35 patients carrying already a MMR mutation and characterized by either a poor clinical phenotype (early age of diagnosis or the diagnosis of multiple cancers) or by a neutral clinical outcome (diagnosis with endometrial cancer only and late in life).

## RESULTS

### Patients

Table 1 shows the clinical features of the women enrolled. All were diagnosed with endometrial cancer between 31–81 years (mean: 53.1 ± 10.7). Subjects belonged to 29 families with a Lynch syndrome mismatch repair (MMR) pathogenic mutation, and all women carried the mutation. These mutations will be referred to as ‘familial MMR mutations’ (Table 1). One familial MMR mutation in *MSH6* (c.3729_3732dupATTA – p.(Phe1245Ilefs*31)) was a founder mutation common to nine subjects belonging to six families.

**Table 1:** Overview of the study population

ID number 12-04-079	Rel.	CLINICAL CHARACTERISTICS							gene	MMR MUTATION				LOE IHC^##^
		Age of EC diagnosis	Type EC	grade EC	stage EC	MSI^$^	Other tumor sites**	cancer phenotype		DNA change	protein change	LOVD ID***	NGS confirmed	
11	D	38	endometrioid	1	1a	I		poor	MSH2	c.244A >T	p.Lys82*	MSH2_00750	YES	na
12	D	46	endometrioid	3	1b	S		poor	MSH2	c.244A >T	p.Lys82*	MSH2_00750	YES	na
16	D	38	na	na	na	na	C-29;C-43	poor	MSH2	c.244A >T	p.Lys82*	MSH2_00750	YES	na
17	E	46	endometrioid	na	na	S		poor	MSH2	c.212–2A >G	splice alteration	MSH2_00072^4^	YES	na
19	E	53	na	na	na	na		neutral	MSH2	c.212–2A >G	splice alteration	MSH2_00072^4^	YES	na
26	F	49	endometrioid	1	1a	na		poor	MSH6	c.1139_1143delATGAG	p.Asp380Alafs*6	MSH6_00833^5^	YES	na
28	F	55	endometrioid	1	1b	na		neutral	MSH6	c.1139_1143delATGAG	p.Asp380Alafs*7	MSH6_00833^5^	YES	na
18	G	44	endometrioid	1	na	I		poor	MLH1	c. 2149_2195dupl	p.His733Asnfs*66	MLH1_00830	NO^1^	na
29	G	39	na	na	na	na	O-na	poor	MLH1	c. 2149_2195dupl	p.His733Asnfs*66	MLH1_00830	NO^1^	na
2	A	52	endometrioid	2	1b	S		neutral	MSH6	c.3729_3732dupATTA	p.Phe1245llefs*31^3^	MSH6_00330	YES	na
3	A	65	serous	3	1c	I	C-59	poor	MSH6	c.3729_3732dupATTA	p.Phe1245llefs*31^3^	MSH6_00330	YES	Y
6^^^	B	51	endometrioid	1	1b	S		neutral	MSH6	c.3729_3732dupATTA	p.Phe1245llefs*31^3^	MSH6_00330	YES	na
7	B	62	endometrioid	3	2	I	C-70;U-81	poor	MSH6	c.3729_3732dupATTA	p.Phe1245llefs*31^3^	MSH6_00330	YES	Y
8	C	54	endometrioid	1	1a	I		neutral	MSH6	c.3729_3732dupATTA	p.Phe1245llefs*31^3^	MSH6_00330	YES	Y
9	C	52	endometrioid	1	1	S	B-na	poor	MSH6	c.3729_3732dupATTA	p.Phe1245llefs*31^3^	MSH6_00330	YES	na
1	-	50	endometrioid	1	2	I		neutral	MSH6	c.3729_3732dupATTA	p.Phe1245llefs*31^3^	MSH6_00330	YES	Y
4	-	57	endometrioid	3	na	I		neutral	MSH6	c.3729_3732dupATTA	p.Phe1245llefs*31^3^	MSH6_00330	YES	Y
5	-	62	endometrioid	1	1b	I		neutral	MSH6	c.3729_3732dupATTA	p.Phe1245llefs*31^3^	MSH6_00330	YES	Y
10	-	40	endometrioid	1	na	na	O-40	poor	MSH6	c.1444 C >T	p.Arg482*	MSH6_00066^6^	YES	na
13	-	81	na	na	na	na	C-59	poor	PMS2	c.989–296_1144+706del	p.Glu330_Glu381del	PMS2_00039	NO^1^	na
14	-	31	na	na	na	na		poor	MSH6	c.3772 C >T	p.Gln1258*	MSH6_00259^7^	YES	na
15^^^	-	49	endometrioid	1	3	S	O-49	poor	MSH6	c.2569_2572del	p.Asp857Phefs10*	MSH6_00326	YES	Y
20	-	56	endometrioid	na	na	S	B-50	poor	MSH6	c.2191C >T	p.Gln731*	MSH6_00092^8^	YES	na
21	-	58	endometrioid	1	1b	I	C-58	poor	MLH1	c.901C >T	p.Gln301*	MLH1_00407	YES	Y
22	-	60	endometrioid	1	3	na		neutral	MSH6	c.2815 C >T	p.Gln939*	MSH6_00465	#	na
23	-	49	endometrioid	1	1b	I		poor	PMS2	c.24–12_107del96	p.Ser8Argfs5*	PMS_00205	NO^1^	Y
24	-	61	endometrioid	3	1b	I	C-56	poor	MSH6	c.4002–22_4002–4del19	splice alteration	MSH6_00335	YES	Y
25	-	44	endometrioid	1	na	I		poor	MSH6	c.3838C >T	pGln1280*	MSH6_00554	#	Y
27	-	40	na	na	na	S		poor	MSH2	c.212-?_366+?del	p.Ala72Phefs*9	MSH2_00076	NO^1^	na
30	-	71	endometrioid	2	3	I		neutral	MSH2	c.646–2A >G	splice alteration	MSH2_00751	NO^2^	na
31	-	50	mixed	3	1b	I		neutral	MSH6	c.2191C >T	p.Gln731*	MSH6_00092	YES	Y
32	-	64	endometrioid	1	1a	S		neutral	MSH6	c.2926_2929dupCGTT	p.Tyr977Serfs*8	non-deposited	YES	Y
33	-	49	endometrioid	1	1b	I		poor	MSH6	c.1804_1805del2	p.Ser602Lysfs*4	non-deposited	YES	Y
34	-	75	endometrioid	1	1b	na	C-na	poor	MSH6	c.3794_3801del8	p.His1266Metfs*6	non-deposited	YES	na
35	-	64	endometrioid	1	1b	I		neutral	MSH6	c.3949_3965del17	p.His1317Ilefs*2	non-deposited	NO^1^	Y
37	-	53	na	na	na	na	C-52	poor	MSH2	c.1203dup	p.Gln402Thrfs*15	MSH2_00896	YES	na
38	-	55	endometrioid	2	1	I		neutral	MSH6	c.3185G >T	p.Cys1062Phe	MSH6_00885	NO^2^	na

Rel.: the same letters indicate a familial relationship.

$ MSI: microsatellite instability in endometrial cancer specimen. S= stable; I = instable.

** C-n = colorectal cancer-age at diagnosis; O-n = ovarian cancer-age at diagnosis; B-n = breast cancer-age at diagnosis; U-n = tumor of urinary tract-age at diagnosis.

*** LOVD ID: mutation is deposited with the indicated number in the *Leiden Open Variation Database*.

# NGS was of poor quality and sample was excluded from further analyses.

## LOE of MMR: loss of expression of MMR protein in endometrial cancer specimen. Y = loss of expression; N = no expression loss.

^ samples were subjected to NGS twice to assess reproducibility.

1 large rearrangements could not be detected by our pipeline.

2 no sufficient coverage of the specific region in this sample.

3 this is a founder mutation.

4–8 Mutations are also deposited in the dbSNP database: 4 = rs267607917; 5 = rs142111387; 6 = rs63750909; 7 = rs63750554; 8 = rs63751442.

na: non-available/non-analyzed.

Eleven women were diagnosed with a second Lynch syndrome tumor (two in the breast, six in the intestine, three in the ovaries). One woman developed colorectal and urinary tract cancers and one was diagnosed twice with colorectal cancer (14 years apart). Additionally, 14 women developed endometrial cancer at the age of 49 or earlier. In the following analyses, women diagnosed with more than one cancer, or with endometrial cancer before 50 (49 or younger) are defined as patients with poor clinical phenotypes (or outcome, *n* = 23), whereas 14 patients who developed only endometrial cancer and after the age of 50 are defined as having a neutral clinical phenotype.

### Estrogen and cancer related 154 gene-panel

The coding sequences and splice regions (10 non-coding nucleotides at each exon end) of 154 genes implied in endometrial physiology and carcinogenesis were explored by Next Generation Sequencing (NGS). Table 2 overviews the captured panel and Supplemental Table S1 gives the complete list of genes and the full set of related references.

**Table 2:** Overview of the panel design of 154 estrogen and cancer associated genes with the variants identified in 35 subjects analyzed

	Capture plan		Variants identified						
			Total	dbSNP deposited*			non-dbSNP deposited		
	No of genes	nt (Kb)	No	Missense + non-sense	Silent	Intron-exon	Missense + non-sense + ins / del	Silent	Intron-exon
**ESTROGEN**	47	101.407	25	9	4	0	11	0	1
*ligand metabolism*	*10*	*13.501*	*3*	*0*	*0*	*0*	*3*	*0*	*0*
*target genes*	*24*	*46.847*	*12*	*6*	*2*	*0*	*3*	*0*	*1*
*responsive genes*	*7*	*10.539*	*1*	*0*	*0*	*0*	*1*	*0*	*0*
*co-regulators*	*6*	*30.52*	*9*	*3*	*2*	*0*	*4*	*0*	*0*
**ONCOGENES**	35	112.56	24	3	5	2	8	6	0
**TUMOUR SUPPRESS**	63	162.306	40	17	9	0	8	4	2
**OTHER**	9	19.395	9	0	3	0	5	0	1
**TOTAL**	**154**	**395.668**	**98**	**29**	**21**	**2**	**32**	**10**	**4**

* variants deposited in the dbSNP database with a MAF <0.001.

The list of all variants is given in Supplemental Table S3.

Estrogens are important physiological regulators in the endometrium and risk factors for cancer [12]. Hence, the capture panel included 47 genes involved in estrogen (and progesterone) signaling, comprising genes controlling the metabolism and degradation of steroids [12], genes directly targeted by the hormone receptors [16–18], genes responsive to steroids [17, 19] and genes encoding for co-regulators controlling the hormone receptor transcriptional activity [16–18]. In addition, 35 oncogenes and 63 tumor suppressors were captured [10, 20–22]. NGS and Quality Control analyses are described as Supplemental Materials (Supplemental Methods, Supplemental Figures S1, 2 and Supplemental Table S2). Thirty-seven DNA samples were sequenced but two were excluded due to low quality (12–04-079_22 and 12–04-079_25, Supplemental Materials and Table 1). As control of NGS and raw data analyses, two samples (12–04-079_6 and 12–04-079_15) were randomly selected and subjected to one additional and independent library preparation and NGS run and they showed 100% concordance between called variants.

### Variant analyses

Among the 35 patient's DNA successfully sequenced, 865 independent variants deposited in dbSNP database were identified and each subject carried on average 245 single nucleotide polymorphisms (SNPs; range 207–270). Common SNPs were excluded from further analyses, but since rare dbSNP variants can be relevant in our search for genetic risk modifiers [23, 24], 52 variants with a minor allele frequency (MAF) lower than 0.001 were retrieved (Table 2). Of these, 28 were missense, one non-sense (stop), 21 were silent and two located in non-coding intron/exon boundaries. In addition, 46 identified variants were not reported in dbSNP, and 34 of these were never deposited before in any database (ExAC, Cosmic, LOVD and HGMD were checked; Table 2). Twenty-nine missense, one frame-shift non-sense, one in frame deletion and one in frame insertion of one amino-acid were identified plus 10 silent variants and four changes located in the non-coding regions flanking an exon (Table 2).

In total, 98 rare variants were identified (dbSNP with MAF <0.001 and non-dbSNP deposited). Considering only variants affecting the amino-acid sequence, there were 57 missense, one amino-acid insertion, one deletion (both in-frame) and two non-sense changes. All variants were heterozygous (Supplemental Table S3).

### Putatively genetic risk modifiers: risk-variants

To predict the functional consequences and potential risk modifying effects, variants were curated using defined criteria [23–25], literature, controlling manually and in combination with bioinformatics tools such as: Alamut (version 2.4, Interactive Biosoftware, including SIFT and PolyPhen-2), Uniprot, PhosphoSitePlus^®^ (for three-dimensional structure and post-translational modifications) [15], NCBI Clinical Variants, ExAC, NHLBI ESP, LOVD, HGMD, Cosmic.

Variants outside the MMR genes were analyzed first. The 98 rare variants reported earlier were considered plus three SNPs with a MAF ≥ 0.001 previously reported as pathogenic or disease related. These were: rs57865060, chromosome position Chr15:75012998:delT, with predicted protein change CYP1B1:p.(Glu229Lys); rs121918303, Chr13:32351535:A > C with predicted protein change RXFP2:p.(Thr222Pro); rs56378716, Chr17:56356502: A > G, with predicted protein change MPO:p.(Met251Thr).

The resulting 101 variants (Supplemental Table S3) were categorized in five groups according to the recommendations of the Dutch and British societies for clinical genetics [24]: categories 1 and 2 = no effect; 3 = variants of unknown significance – VUS; 4 = likely pathogenic; 5 = pathogenic. Silent and non-coding variants were categorized as 1 (none was predicted to affect splicing or other regulatory functions). Conservative amino acid changes were considered unlikely to affect the protein function (category 2), with the exclusion of four variants that had been associated with disease or were predicted to have damaging effects (Alamut prediction tool), which were categorized as 3. The remaining class 3 variants were all non-conservative protein changes. Eight variants were in category 4, four of which were pathogenic (class 5) for disorders other than endometrial cancer, hence they were considered as class 4 in the present study. Two independent investigators (RA and BMJ, of which BMJ is a Clinical Laboratory Geneticist with expertise in diagnostics and oncogenetics) performed this curation blindly for each other's results. Supplemental Table S3 gives the curation details and the complete variant list.

Since genetic risk modifiers have modest effects (see discussion), all variants categorized as 3 (provided the presence of a non-conservative amino-acid substitution) or higher were considered and resulted in 54 candidate risk-modifiers. Such variants, referred to as ‘risk-variants’ from now on, were further characterized for the following features (Supplemental Table S3): *i)* the presence of cancer somatic (not silent) mutations in the same protein region (i.e. max +/−3 amino acids from the identified risk-variant; Cosmic database). This was considered as an indication that changes in the specific protein region could affect function and predisposition to cancer; *ii)* the interspecies protein domain conservation; *iii)* the presence of the same risk-variant among relatives; *iv)* the occurrence of the risk-variant in relevant protein domains; *v)* the predicted consequences based on Alamut, protein in silico analysis, literature mining or the occurrence of other pathogenic variants/SNPs in the same region; *vi)* we excluded that risk-variants could co-segregate with the familial MMR mutation by verifying their localization on separate chromosomes (Supplemental Table S3).

The majority of the patients carried one (or more) risk-variant in estrogen related genes (19 out of 35 patients, 54%) and 18 risk-variants were unique (i.e. the same variants occurring in relatives were counted only once: *n* = 5). Risk-variants in tumor suppressors were observed in 18 patients (49%), and 25 were unique. One (or more) oncogene contained nucleotide changes in nine patients (26%), and nine were unique.

**Figure 1:**
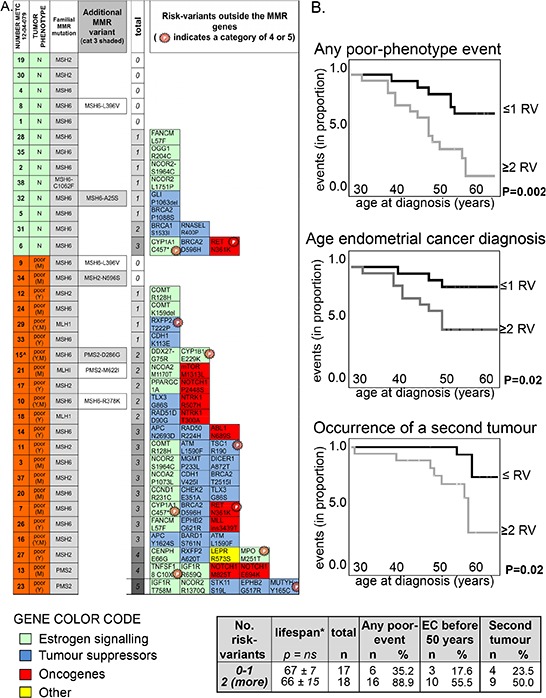
The number of class 3 (or more) risk-variants correlates with poor clinical phenotypes **A.** Heat map showing the presence of class 3 (or more) risk-variants in each subject with neutral phenotype (green, N) or with a poor phenotype (orange, poor; Y = young age of endometrial cancer diagnosis; M = cancer at multiple sites). The familial MMR mutations (all non-sense but the one carried by subject 12–04-079_38) are indicated (3^rd^ column). ‘Additional MRR variants’ (4^th^ column) reports the six rare variants (MAF < 0.001) found in MMR genes among seven subjects. In particular, MSH6:p.(Arg378Lys) in subject 12–04-079_10 has been already described to be in linkage with the familial MSH6:p.(Arg482*) mutation detected in the patient [39]. ‘Total’ (5^th^ column) refers to the total number of risk-variants outside the MMR genes identified in each patient. The specific risk-variants are reported at the right as gene name and predicted amino-acid change (for full chromosomal coordinates/nucleotide change, see Supplemental Table S3), together with a color code that indicates the gene function. Variants previously reported as (likely) pathogenic (categories 4 and 5) are labeled with a starred ‘P’. **B.** Kaplan-Meier curves showing the occurrence of any poor-clinical phenotype feature (*Top chart*; first events were only considered for statistic), of the diagnosis of endometrial cancer before 50 (*Middle chart*), or of the occurrence of a second tumor (*Bottom chart*; first events were only considered for statistic) in subjects carrying a maximum of one risk-variant (indicated as RV) compared to subjects carrying two or more risk-variants outside the MMR gene. The statistical p for significance is computed by Log Rank (Mantel-Cox, Test of equality of survival distributions for the different levels of risk-variants; SPSS).

MMR genes also contained three risk-variants beside the familial mutation and these have not been computed in the list of 101 variants given so far (Figure 1A). Two variants, one in *PMS2* -rs116788608, Chr7:6035211:T > C, with predicted protein change PMS2:p.(Asp286Gly)- and the second in *MSH2* - rs41295288, Chr2:47702191:A > G with predicted p.(Asp596Ser)- were on other chromosomes than the familial MMR mutation (*MSH6*) of these subjects. In contrast, *MSH6* variant with predicted protein change p.(Ala25Ser) (rs267608026, Chr2:48010445:G > T) in patient 12–04-079_32 could co-segregate with the *MSH6* familial mutation carried by this subject. Besides these three risk-variants, three additional variants in MMR genes with MAF < 0.001 were classified in category 2 (Figure 1A).

### Risk-variants are associated with poor clinical phenotypes

The distribution of MMR pathogenic mutations did not vary between women with poor and neutral clinical phenotypes (Table 3), but patients with poor clinical phenotypes carried more frequently one or more risk-variants outside the MMR genes compared with women with a neutral phenotype (Table 3 and Figure 1A).

**Table 3:** distribution of familial MMR mutations and risk-variants outside the MMR genes (class-3 or more, i.e. putatively risk modifiers) among patients with neutral and poor clinical phenotypes

	Total subjects	Neutral tumor phenotype		Poor tumor phenotype		
		No.		No.		*p*-value
		14**		23**		
	Lifespan*	64.2 ± 6.0		66.8 ± 14.1		ns^#^
**FAMILIAL MMR MUTATIONS**		**No.**	**%**	**No.**	**%**	***p*-value**
	***MLH1***	0	0.0	3	13.6	ns^##*^
	***MSH2***	2	15.4	6	27.3	
	***MSH6***	12	92.3	12	54.5	
	***PMS2***	0	0.0	2	9.1	
PRESENCE OF ADDITIONAL RISK-VARIANTS***	***no variant***	5	38.5	2	9.1	0.0471^##@^
	***one variant***	6	46.2	4	18.2	
	***two variants***	1	7.7	5	22.7	
	***three variants***	1	7.7	8	36.4	
	***four variants***	0	0.0	2	9.1	
	***five variants***	0	0.0	1	4.5	
	***0 or 1 variant***	11	84.6	6	27.3	0.0016^###^
	***2 or more var.***	2	15.4	16	72.7	

* age of subjects in 2013 was used as lifespan (mean age ± standard deviation in years).

** 13 and 22 subjects were successfully analyzed by NGS.

*** risk-variants, i.e. class-3 or higher, in genes other than the MMR were considered.

# *T*-test to compare mean values.

##* Chi square, with 3 degrees of freedom.

##@ Chi square, with 5 degrees of freedom, Pearson's: 11.225.

### Fisher exact test, two sided *p*-value.

*ns* = non-significant.

Cox-regression analysis confirmed that increasing number of risk-variants was associated with a poor clinical phenotype (Supplemental Figure S3). In these Kaplan-Meier analyses the curves of subjects with no or one risk-variant overlap with each other and are separated from the curves of subjects with two, three or more than four risk-variants. Table 3 (rows at the bottom) also shows a highest statistical significance by combining these two groups. Because of this net distinction between subjects carrying no or one risk-variant *versus* women carrying more than two risk-variants, in subsequent Kaplan-Meier cox-regression analyses we combined patients in these two groups (Figure 1B). The presence of two or more risk-variants beside the familial MMR mutation was associated with the occurrence of poor clinical features. As defined earlier, poor clinical features were either a diagnosis of endometrial cancer at a young age (<50), or the onset of multiple cancers. Subsequently, these two characteristics were assessed separately and the presence of two or more risk-variants was associated with both (Figure 1B). The lifespan among patients with neutral and poor clinical phenotypes, extrapolated at 2013 (the date of last update of our database), did not vary significantly between groups (Table 3, Figure 1B and Supplemental Figure S3).

To exclude that multiple risk-variants could co-occur because of co-segregation, we verified that all risk-variants in one subject were located on separate chromosomes. This was confirmed for 16 out of the 18 subjects. Subject 12–04-079_14 carried risk-variants Chr5:112179368:A > G (APC:p.(Asn2693Asp)) and Chr5:131915673:G > A (RAD50:p.(Arg224His)) both on chromosome 5 (at a distance of about 90 Mb). A second patient (12–04-079_13) carried two risk-variants in the same gene (*NOTCH1*). Results did not change by considering these four risk-variants as two co-segregating haplotypes.

Three possible risk-variants (category 3 or higher) were identified in MMR genes (grey shaded in Figure 1A), which were not considered in the aforementioned analyses because a linkage with the MMR familial mutation could not be excluded. Nevertheless, when these risk-variants were included, results did not change. In fact, two risk-variants that lied certainly in other chromosomes than the MMR familial mutation – Chr7:6035211:T > C, predicted protein change PMS2:p.(Asp286Gly) and Chr2:47702191:A > G with predicted MSH2:p(Asp596Ser)- occurred in patients with a poor clinical phenotype, whereas risk-variant Chr2:48010445:G > T, with predicted change MSH6:p.(Ala25Ser), which could be in linkage with the familial mutation, occurred in subject 12–04-079_32 having a neutral clinical phenotype.

Among 15 subjects there were some 1^st^ and 2^nd^ degree parental relationships and they belonged to 7 families. Among the five families in which members had distinct neutral or poor clinical phenotypes, there were three families (A, E and F) in which the woman with poor phenotype carried two or more risk-variants and carried more risk-variants than the relative with a neutral phenotype (Supplemental Figure S4). In family B, both members carried three risk-variants but the woman with neutral phenotype was significantly younger than the women with poor phenotype, and the occurrence of a poor phenotype later in life cannot be excluded.

### Protein characterization in tumors

Immunohistochemistry was used to further characterize the protein expression in a limited number of endometrial tumor specimens carrying the risk-variants (formalin-fixed-paraffin-embedded, FFPE). Risk-variants and relative predicted protein change were: Chr10:43604498:C > A, with predicted protein change RET:p.(Asn361Lys); Chr15:75012998:delT, CYP1A1:p.(Cys457*); Chr15: 99465448:C > T, IGF1R:p.(Thr758Met), Chr12:124821523:G > C, NCOR2:p.(Ser1964Cys); Chr22: 19951274:delAAG, COMT:p.(Lys159del); Chr1:11264 625:T > G, mTOR:p.(Met1313Leu). We will refer from now on to these risk-variants using their predicted protein changes. Results on protein expression in the Lynch tumors were compared with a panel of sporadic endometrial cancers (*n* = 7) and post-menopausal healthy controls (*n* = 5). The presence of the risk-variant-allele in the Lynch tumors was confirmed by Sanger sequence analyses in 12 samples and none showed loss of heterozygosity (LOH, listed in Supplemental Table S4).

Since we found a substantial number or risk-variants mapping in the estrogen signaling, the expression levels of the estrogen receptor (ER-α) and the Ser-167-phosphorylated form, induced by growth factors (including mTOR, IGF1R and RET) and associated with ligand-independent activation (26–28), were assessed. ER-α and phospho-Ser-167-ER-α levels varied between samples, with highest expression in non-cancerous endometrium (both from healthy controls and normal tissues adjacent to cancer cells, i.e. peri-tumoural tissue), intermediate levels in well-differentiated cancers and lowest levels in high-grade lesions (Supplemental Figure S5).

**Figure 2:**
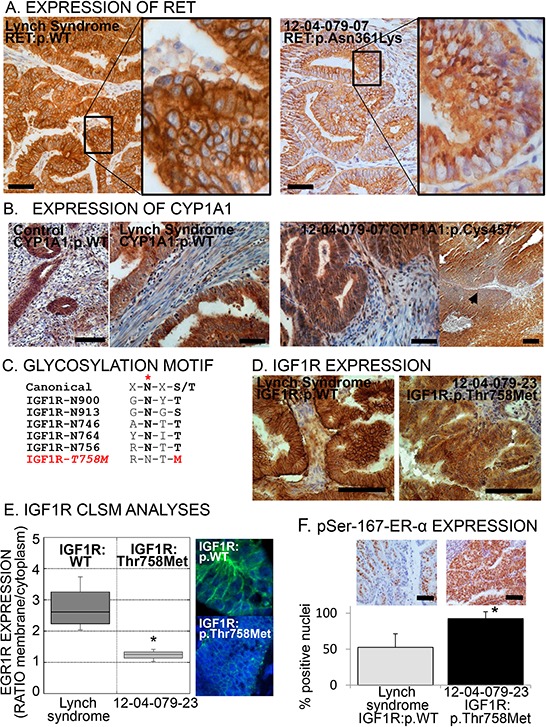
Aberrant expression or localization of RET, CYP1A1 and IGF1R in specimens carrying the respective risk-variants **A.** Immunohistochemical images of tumors bearing the common allele (WT) of RET or the risk-variant RET:p.(Asn631Lys). Note the punctuated cytoplasmic pattern in the risk-variant carrying tumor indicative of trapping of the receptor inside the cell. Scale bar = 100 μm. **B.** Immunohistochemical images of endometrial specimens bearing the common allele (WT) of CYP1A1 (control post-menopausal endometrium and Lynch syndrome endometrial cancer: left images). Right images, CYP1A1 expression on tumor specimen bearing the p.(Cys457*) risk-variant, showing one area with loss of expression (arrowhead). Scale bar = 100 μm. **C.** Canonical consensus and the five glycosylation sequences (glycosylation site indicated by red asterisk) in the IGF1R plus the consequent disruption of this consensus caused by risk-variant IGF1R:p.(Thr768Met). **D.** Immunohistochemical images of tumor specimens with common allele (WT) of IGF1R or with risk-variant p.(Thr758Met). Scale bar = 100 μm. **E.** Representative confocal laser scanning microscopic (CLSM) images and fluorescence quantification of the membrane/cytoplasmic fractions of IGF1R in tumor with common allele or with p.(Thr758Met) IGF1R. The box plot represents the distribution of four independent samples for common allele and four areas of sample 12–04-079–23 [carrying the p.(Thr758Met) risk-variant]. Asterisk indicates a *p*-value < 0.05, Wilcoxon-Mann-Whitney Rank Sum Test. **F.** Representative immunohistochemical images (top) and quantification of the percentage of nuclei positive to phospho-Ser-167-ER-α among endometrial cancer with wild type IGF1R (the grey bar indicates the mean ± SD of seven Lynch syndrome tumor specimens) and with IFG1R:p.(Thr758Met) (black bar, mean ± SD of four independent areas of the tumor from subject 12–04-079–23, bearing the variant). Asterisk indicates a *p*-value < 0.05 compared to common allele (*t*-test). Scale bar = 100 μm.

The expression of NCOR2, CYP1A1 and COMT, involved in the estrogen signaling (see discussion), and the oncogenes mTOR, RET and IGF1R was high in normal and cancerous endometrium and varied between samples (Figure 2 and Supplemental Figures S5 and S6).

The identified risk-variant p.(Asn361Lys) in the RET tyrosine kinase receptor has been previously described in Hirschsprung disease. Asn361 is a N-linked glycosylation site necessary for correct membrane localization of the receptor and mutated RET remains trapped in the endoplasmic reticulum (29). Immunohistochemistry on sample 12–04-079_7, carrying RET:p.(Asn361Lys), clearly shows an aberrant RET cytoplasmic localization compared with controls where RET is located at the plasma membrane (Figure 2A).

The frame-shift change in CYP1A1 at position 457 is predicted to replace the cysteine heme-binding site (30) with a stop codon thus disrupting the catalytic site and causing premature protein termination. Partial and localized loss of CYP1A1 expression was observed in the tumor carrying this risk-variant (Figure 2B).

Risk-variant p.(Thr758Met) in the tyrosine kinase receptor IGF1R is predicted to abolish the N-linked glycosylation at residue Asn756 (Figure 2C). Glycosylation is needed for correct receptor processing, membrane localization and signal transduction (31). Specimen 12–04-079_23 carrying this risk-variant showed a decreased membrane immuno-localization of the IGF1R (Figure 2D), which was confirmed with confocal laser scanning microscopy (CLSM; Figure 2E). This observation was concomitant with the highest percentage of phospho-Ser-167-ER-α staining (Figure 2F), one of the downstream target of the IGF1R kinase activity, suggestive of a sustained intracellular activation.

## DISCUSSION

Genome-wide approaches [5, 6, 32] have identified modifiers of the cancer risk conferred by pathogenic Lynch syndrome mutations, but additional genetic modifiers are predicted to exist. For instance, genome-wide studies cannot detect rare variants [33] whose risk modifying contribution remains largely undetermined. To identify rare genetic modifiers of the risk of Lynch syndrome-related endometrial cancer, we have screened a panel of 154 genes among a cohort of 35 Lynch syndrome related endometrial cancer patients. Fifty-four variants with putative risk modifying action were selected (risk-variants), which occurred in 40 genes and the presence of two or more risk-variants was associated with poor clinical phenotypes (Figure 1). This result is in line with genome-wide studies that have shown that risk modifying alleles exert larger risks in carriers of multiple variants compared with single-variant carriers [6].

The rationale used to design the 154 gene-panel and the criteria used to select putative risk modifiers derived from recent understandings in cancer biology. Cancer somatic mutation landscapes have shown that while somatic driver mutations inactivate tumor suppressors or activate oncogenes, germ-line predisposing variants have more subtle effects and do not necessarily give a direct growth advantage or cancer phenotype [11]. For these reasons, the designed gene-panel included not only cancer associated-genes but also genes that are broadly involved in the endometrial physiology, like those encoding for proteins controlling the estrogen signaling. In line with this model suggesting a distinction between germ-line predisposing *versus* somatic driver mutations [11], no (germ-line) risk-variant was identified among the genes that are most frequently mutated at the somatic level in endometrial cancer, such as *PTEN*, *PIK3CA*, *CTNNB1*, *KRAS* (all included in the panel). When endometrial cancer somatic mutation landscape (COSMIC database [10]) was aligned to the risk modifiers identified in the present study, *MTOR* was the first common gene ranking 130^th^ among somatic mutations. *STK11* and *ATM* appeared as risk modifiers in our screen and are frequently mutated somatically [9] but not in endometrial cancer (COSMIC database [10]).

Well-established criteria were used to cluster variants in categories 1–5 [23–25, 34]. In addition, we considered that genetic modifiers are expected to have little or no influence in the general population (modest effect-size), while influencing the cancer-risk in predisposed subjects, i.e. in subjects carrying a penetrant cancer mutation [2]. Hence, we considered as putative risk modifiers all variants having a likely-pathogenic or pathogenic effect (category 4/5) but also those with ‘unclear significance’ (category 3), provided that the were missense leading to non-conservative amino-acid substitution. In this context, the recognition of the role of rare missense changes in cancer susceptibility is growing [35]. Lower categories (2 and 1, unlikely to have any effect at the protein level) were excluded.

Genes such as *NOTCH1* (cell-fate determination), *NCOR2* and *NCOA2* (controlling the activity of ER-α), *COMT* (detoxification pathway and estrogen degradation), and classical oncogenes (the tyrosine kinase receptors *NTRK1* and *IGF1R*) or tumor suppressors (*APC*, *BRCA2*, *EPHB2* and *CDH1*, the latter being already identified as a risk modifier locus in genome-wide studies [6]) carried more than one independent risk-variant, indicating that they are hit frequently. In addition, the pathways where risk-variants were found underscored some signalings in which genetic modifier can be found: the *STK11*/*TSC1*/*mTOR* cascade; the estrogen signaling at ER-α transcriptional control (*NCOR2*, *NCOA2* - both with multiple risk-variants *-* and *PPARGC1A*); or at ligand metabolism level, with risk-variants identified in *CYP1A1*, *COMT* and *CYP1B1*, phase I and II detoxifying enzymes degrading also estrogens via the formation of catechol-compounds. The CYP1A1 locus was reported previously as risk modifier for Lynch syndrome colorectal cancer, although data were not confirmed in independent studies [5] and common variants in *CYP1A1* and in other genes controlling the estrogen metabolism have been associated with sporadic endometrial cancer [36].

Although it was not possible to assess the segregation of the identified risk-variants with the disease within the families in our cohort (DNA from unaffected relatives with matched life-span was not available), poorer phenotypes tended to segregate with a higher number of risk-variants among the small number of patient-relatives included in our cohort (Supplemental Figure S4).

Further immunohistochemical characterization of a limited number of risk-variants showed association with protein changes. The estrogen degrading protein CYP1A1 showed localized loss of expression in the tumor carrying CYP1A1:(p.Cys457*). Tyrosine receptors including RET and IGF1R have important crosstalk's with the estrogen signaling and are relevant to the endometrial physiology [28, 37]. Variant RET:p.(Ans361Lys) impairs RET correct membrane localization [29], and was indeed associated with aberrant cytoplasmic immunoreactivity in this study. Glycosylation of IGF1R is reported to be important for cellular localization, response to anti-IGF therapy and signaling activation [31]. Variant IGF1R:p.Thr758Met, predicted to disrupt one N-linked glycosylation site, was associated with diminished membrane IGF1R localization and increased phosphorylation of ER-α, one of the downstream IGF1R kinase targets. The *IGF* gene locus (coding for the ligand of IGF1R) has been reported as a genetic risk modifier for Lynch syndrome [5].

In conclusion, we propose a gene-panel that contains potential genetic risk modifiers of Lynch syndrome related endometrial cancer in our population. Risk-variants that are likely pathogenic have been identified and assessing their segregation with the disease phenotypes can be relevant to the family members and the subjects assisted at our center.

For implications outside the families of the present investigation, the development of gene-panels in diagnostics is technically desirable compared to complete genome sequencing because it allows achieving sufficient coverage, cost effectiveness and simplicity in analyses [11, 23]. Therefore, it is intriguing to validate these data in independent populations and examine how this panel will translate to other ethnicities. The fact that a number of risk modifiers identified here correspond to loci that were previously characterized in genome-wide investigations is encouraging. Additionally, it is possible to improve our panel design by including genes recently identified as risk modifiers in genome-wide studies [6, 38].

## MATERIALS AND METHODS

### Ethical statement

Investigation has been conducted in accordance with the ethical standards, according to the Declaration of Helsinki and according to national and international guidelines. Protocols have been approved by the authors' institutional medical ethical committee (see below for details).

### Patient population

Thirty-seven Dutch Caucasian women from 29 families who developed hereditary endometrial cancer and carried a germ-line mismatch repair (MMR) gene mutation were enrolled (Table 1). Women were counseled at the Maastricht or Leiden Medical Centers (2011–2013) because of a Lynch syndrome family history, a Lynch syndrome mutation or a Lynch Syndrome diagnosis (Bethesda criteria). Genomic DNA was used for Next Generation Sequencing (NGS) analyses. All women had given consent to use their DNA for research. The local medical ethical committee approved the protocol (METC 12–04-079).

Archival formalin-fixed-paraffin-embedded (FFPE) tissues of 12 women's tumor specimen were retrieved from the hospitals that performed the hysterectomy and they were used for DNA analyses, loss of heterozygosity (LOH; *n* = 12) and histology/immunohistochemistry (*n* = 8). Seven sporadic endometrial cancers and five post menopausal healthy controls were randomly selected from our tissue-bank [12] and used as control for immunohistochemistry (local medical ethical committee tissue-bank protocol approval: METC-14–04-003).

### Gene-panel and NGS

Coding sequences plus intron/exon boundaries of 2012 regions from 154 genes (396kB; Table 2, Supplemental Table S1) and 57 off-target control regions (84kB) were designed and captured with Haloplex platform (Agilent Technologies, Ratingen, Germany). Illumina HiSeq NGS (Illumina, San Diego, USA) was used for sequencing and reads were aligned against the reference genome (GRCh37/hg19). Variants were called using NextGene (Softgenetics, State College, USA), SureCall software packages (Agilent Technologies, Ratingen, Germany) plus manual checking, which resulted in 0% false positives (confirmed by Sanger analyses) and in the successful detection of all re- sequenced pathogenic MMR mutations suitable to be detected (Supplemental Methods). Sanger sequencing was performed using BigDye Termination v.1.1. (Life Technologies, Bleiswijk, Netherlands). Detailed descriptions of the gene-panel design, capture, library preparation, NGS, variant calling and quality controls are given in Supplemental Methods. The dbSNP142 - The National Center for Biotechnology Information (NCBI) was used to identify deposited polymorphisms and variants.

### Immunohistochemistry, confocal laser scanning microscopy (CLSM) and LOH

Standard protocols [12, 13] and antibody manufacturer's instructions were used for immunohistochemistry. Antigens were retrieved with tris-EDTA buffer. Antibodies used: estrogen receptor-α (ERα; monoclonal D-5, 1:100; Dako, Glostrup, Denmark), IGF1R (1:400; monoclonal; Novus Biologicals, Littleton, USA), CYP1A1 (1:1000; polyclonal; Sigma-Aldrich, St. Louis, USA), COMT (1:100; polyclonal; Sigma-Aldrich, St. Louis, USA), Phospho-ERα ser167 (1:50; monoclonal; Cell Signaling Technology, Danvers, USA), RET (1:500; polyclonal; Sigma-Aldrich, St. Louis, USA). Chemate Envision and 3,3-diaminobenzidine (Dako, Glostrup, Denmark) were used to visualize antibody binding. Immunoscores were assessed by two independent investigators (DB and RA) as described [12]. Protocols for SMRT, Phospho-S6 Ribosomal protein-ser235/236 and mTOR staining are given in Supplemental Figure S6.

Confocal laser scanning microscopy (CLSM) was performed on 4 μm thick FFPE tissue sections. Anti IGF1R (1:250; monoclonal; Novus Biologicals, Littleton, U.S.A.) followed by rabbit-α-mouse IgG-FITC (1:100, Dako, Glostrup, Denmark) were used. CLSM was performed with Leica CTR 4000/CTC SPE and the Application Suite/advance fluorescence software (Leica Microsystems B.V., Rijswijk, Netherlands). Image-J program (v 1.48, National Institutes of Health, USA) was used for image analyses.

For LOH, an expert pathologist (VdVKK) identified the tumor regions from histological sections of FFPE materials, which were resected for genomic DNA isolation (QIAamp DNA FFPE tissue kit, Leusden, Netherlands) and Sanger sequencing. At least two separate tumor regions were analyzed.

### Statistical analyses

Kaleidograph (v 4.1, Synergy Software, Reading, USA), SPSS (v 22, IBM Corporation, Armonk, USA) and the online Simple Interactive Statistical Analysis (SISA) were used as indicated in the text.

### URLs

#### Gene browsers

Ensembl Genome Browser: http://www.ensembl.org; NCBI: http://ncbi.nlm.nih.gov; University of California Santa Cruz: UCSC Genome Browser: http://genome.ucsc.edu.

#### Variant analyses

dbSNP database Human Build 142: http://ncbi.nlm.nih.gov; Exome Aggregation Consortium: ExAC, v 0.3, (Cambridge, MA, USA; February 2015): http://exac.broadinstitute.org/; Exome Variant Server, NHLBI GO Exome Sequencing Project (ESP, release ESP6500SI-V2, March-2015), Seattle, USA: http://evs.gs.washington.edu/EVS; Leiden Open Variation Database: LOVD 3.0: lovd.nl; The Human Gene Mutation Database: HGMD (Cardiff University): http://www.hgmd.cf.ac.uk; Genome of Netherlands project, release 5: http://www.nlgenome.nl [14]; Catalogue Of Somatic Mutations In Cancer: Cosmic (Sanger Institute): http://cancer.sanger.ac.uk; PhosphoSitePlus^®^: http://www.phosphosite.org [15].

#### Gene ontology, expression information

NCBI: http://ncbi.nlm.nih.gov; UniProt Knowledgebase: http://www.uniprot.org/uniprot; GeneCards (v 3.12.354 March 2015, Weizmann Institute of Science): http://www.genecards.org.

#### Statistics

SISA: http://www.quantitativeskills.com/sisa/.

## SUPPLEMENTARY DATA, REFERENCES, TABLES AND FIGURES










