# Supplementary material for: Germ-line variants identified by next generation sequencing in a panel of estrogen and cancer associated genes correlate with poor clinical outcome in Lynch syndrome patients
Source: Oncotarget. 2015 Oct 22;6(38):41108–22. doi: 10.18632/oncotarget.5694 (PMC4747393; doi:10.18632/oncotarget.5694)
Supplement: Supplementary file 1 [file oncotarget-06-41108-s001.pdf]

# SUPPLEMENTARY DATA

## Customized gene-panel

A panel of 154 endometrial physiology and cancer related genes was compiled based on literature and own experience [1–9]. Genes involved in estrogen and progesterone signaling, involved in the metabolism of steroid hormones plus oncogenes and tumor suppressors were included. For the complete gene-panel description and full set of references see Supplemental Table S1.

The coding sequences plus intron/exon boundaries based on GenBank and CCDS records were captured (The National Center for Biotechnology). Captured sequences were designed with Haloplex Design Wizard Tool (Agilent Technologies, Ratingen, Germany) using Illumina100 as library type. The designed panel consisted of 396kB from 2012 target regions. As control for off target DNA, 57 additional regions mapping in introns or inter-genic DNA sequences were captured as well (84kB). Parameters were optimized to the latest targeted exome capture platform Haloplex (Agilent Technologies, Ratingen, Germany) and Illumina HiSeq NGS (Illumina, San Diego, CA, USA).

## Next Generation Sequencing - NGS

Genomic DNA was quantified, assessed for quality by Qubit measurement (Life Technologies, Bleiswijk, Netherlands) and for degradation on 1.5% agarose gel. As positive control for the sequencing reactions, the gDNA-control from Agilent was used, and consisted of material from a single Caucasian subject isolated from an early passage lymphoblastic cell line. Target DNA sequences were captured with Haloplex Target Enrichment System (Agilent Technologies, Ratingen, Germany) and barcoded libraries were made according to the manufacturer's protocol. Library quality and DNA content were evaluated on a 2100 Bioanalyzer using the High Sensitivity DNA Kit (Agilent Technologies, Ratingen, Germany). Library clusters were generated using the TruSeq® PE Cluster Kit V3 according to the manufacturer's protocol (Illumina, San Diego, CA, USA) and the paired-end library was sequenced in 2 × 100 cycles paired-end sequencing by synthesis (SBS) using a TruSeq™ SBS Kit v3 on a HiSeq2000 (Illumina, San Diego, CA, USA). Base calling was done by using Casava 1.8 (Illumina, San Diego, CA, USA) and sequence reads were aligned against the human reference genome assembly GRCh37/hg19 (UCSC Genome Browser on Human Feb. 2009). Total target region size was 480052 base pairs (bp) and after sequencing a total of covered target regions of 98.4% could be analyzed (472542 bp).

## Quality assessment of sequencing reactions

Quality Control (QC) analysis on coverage was performed by Surecall (Supplemental Figure S1). A threshold for read depth was set of at least 50 times, and for coverage at 90% of the original regions of interest. Two samples did not match these criteria and were excluded (12–04-079_22 and 12–04-079_25, Supplemental Figure S1 and Table I). Sample 12–04-079_31 was of borderline sufficient quality with a depth of 50 times for about 80% of the regions of interest. This sample was retained because the familial MMR mutation could be retrieved and Sanger sequencing confirmed the validity of the additional variants found. The remaining 34 samples were covered more than 50 times in every nucleotide position, over 95% of the region of interests were covered and could be analyzed. Each region was covered on average 2309 times. Supplemental Figure S2 gives the average coverage per region in our samples, and Supplemental Table S2 reports the coverage information per patient per region of interest.

## Variant calling and validation

NextGene software package (Softgenetics, State College, PA, USA) on unique reads with basic settings and SureCall (Agilent Technologies, Ratingen, Germany) were used for raw data analyses. Alignment and variant calling were performed on unique reads with default settings, using a filter set on the region of interest file provided by the Agilent-Haloplex report. To adjust the settings for variant calling, over 70 variants were analyzed by Sanger sequencing (not shown). After this preliminary optimization of variant calling, we further optimized the variant calling pipeline by integrating results from Nextgene and SureCall software's. This strategy resulted in a highly stringent selection of variants with low false positive rate. To further decrease the false positive rate, called variants were manually controlled by excluding those occurring at the 5′or 3′ prime end of the sequence reads and those having an insufficient quality (Phred score less than 25). This pipeline was suitable to call single or few bases aberrations (substitutions, small insertions and deletions) and gave 0% false positive as confirmed by additional Sanger analyses (Supplemental Table S4, column '*number validated samples*').

Among the previously sequenced (at genetic counseling) pathogenic mutations in MMR genes, 12 out of the 14 familial MMR mutations consisting of few nucleotide alterations were retrieved. These mutations were distributed in 27 subjects and the presence was confirmed in all (see also Table I). Two out of 14 familial MMR mutations consisting of few nucleotide alterations

could not be retrieved because located on regions that were poorly covered in the two DNA carrier samples (12–04-079_30 and 12–04-079_38). Beside these 14 mutations, five additional familial MMR mutations were identified at counseling in six samples (12–04-079_13, 18, 29, 23, 27 and 35) but they consisted of relatively large chromosomal rearrangement that could not be retrieved by our pipeline (Table I).

In addition, among the variants outside the MMR genes, 19 that were non-deposited in the dbSNP database were selected for Sanger sequencing and their presence was validated in all 21 subjects carrying the variant. Also, the presence of seven identified polymorphisms deposited in dbSNP was validated in the 22 subjects' DNA carrying the polymorphic allele.

Sanger sequencing was performed using the BigDye Termination v.1.1. (Life Technologies, Bleiswijk, Netherlands) as recommended. Prior to sequence reactions and analyses, DNA regions were amplified by PCR (Taq polymerase, Fermentas, Burlington, CA, USA) using primers listed in Supplemental Table S4 (forward primers were used for Sanger sequence reactions).

## REFERENCES

1. Cornel KM, Kruitwagen RF, Delvoux B, Visconti L, Van de Vijver KK, Day JM, et al. Overexpression of 17beta-Hydroxysteroid Dehydrogenase Type 1 Increases the Exposure of Endometrial Cancer to 17beta-Estradiol. J Clin Endocrinol Metab. 2012; 97:E591–601.
2. Johnson AB, O'Malley BW. ERasing breast cancer resistance through the kinome. Nature medicine. 2011; 17:660–1.
3. Romano A, Adriaens M, Kuenen S, Delvoux B, Dunselman G, Evelo C, et al. Identification of novel ER-[alpha] target genes in breast cancer cells: Gene- and cellselective co-regulator recruitment at target promoters determines the response to 17[beta]-estradiol and tamoxifen. Mol Cell Endocrinol. 2010; 314:90–100.
4. Zwart W, Theodorou V, Kok M, Canisius S, Linn S, Carroll JS. Oestrogen receptor-co-factor-chromatin specificity in the transcriptional regulation of breast cancer. Embo J. 2011; 30:4764–76.
5. Lawrence MS, Stojanov P, Mermel CH, Robinson JT, Garraway LA, Golub TR, et al. Discovery and saturation analysis of cancer genes across 21 tumour types. Nature. 2014; 505:495–501.
6. Pritchard CC, Smith C, Salipante SJ, Lee MK, Thornton AM, Nord AS, et al. ColoSeq provides comprehensive lynch and polyposis syndrome mutational analysis using massively parallel sequencing. The Journal of molecular diagnostics: JMD. 2012; 14:357–66.
7. Tamborero D, Gonzalez-Perez A, Perez-Llamas C, Deu-Pons J, Kandoth C, Reimand J, et al. Comprehensive identification of mutational cancer driver genes across 12 tumor types. Scientific reports. 2013; 3:2650.
8. Salvesen HB, Carter SL, Mannelqvist M, Dutt A, Getz G, Stefansson IM, et al. Integrated genomic profiling of endometrial carcinoma associates aggressive tumors with indicators of PI3 kinase activation. Proc Natl Acad Sci U S A. 2009; 106:4834–9.
9. Groothuis PG, Dassen HH, Romano A, Punyadeera C. Estrogen and the endometrium: lessons learned from gene expression profiling in rodents and human. Hum Reprod Update. 2007; 13:405–17.

**Supplementary Table S1: List of captured genes**

## Supplementary Table S2: Coverage data per region per sample

**Supplementary Table S3: List of all selected variants (outside MMR genes) identified in 35 subjects**

**Supplementary Table S4: List of primers used for Sanger sequencing analyses**

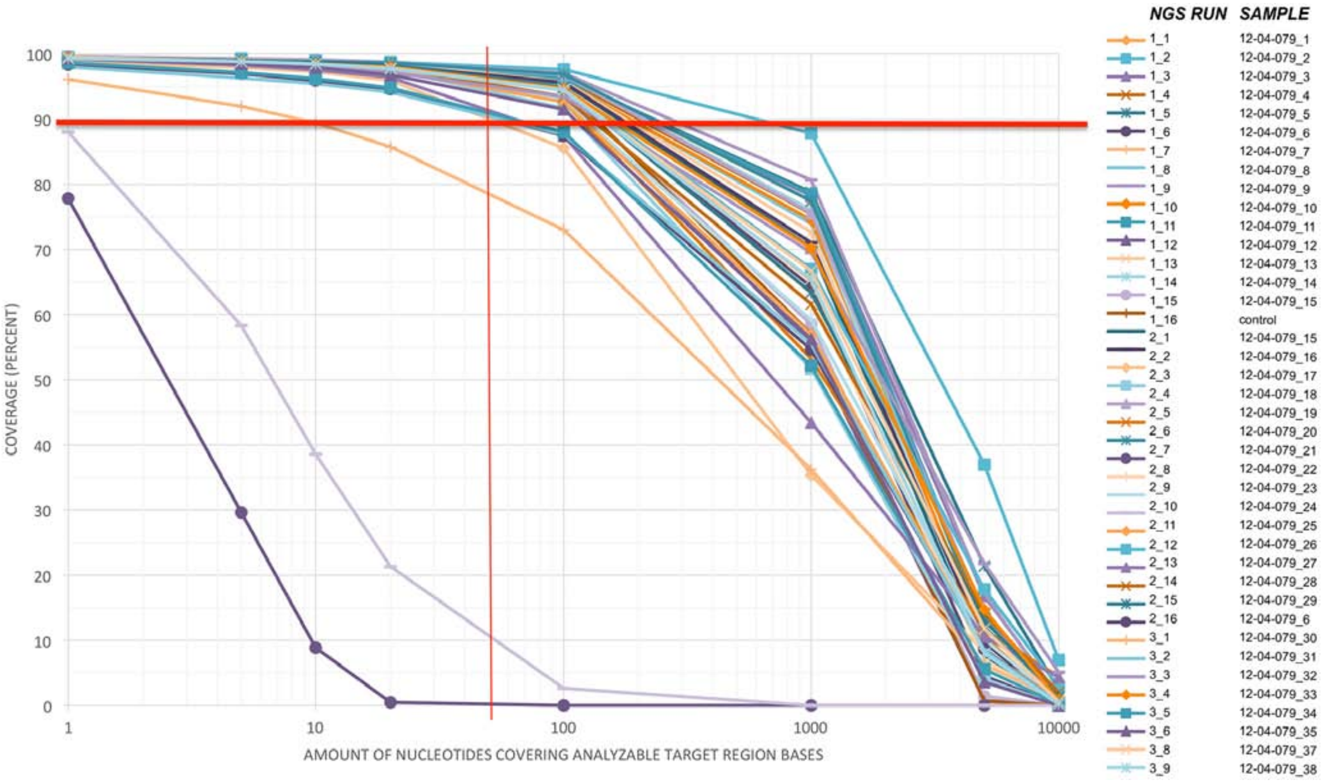


**Supplementary Figure S1: Quality control.** Quality Control analysis on coverage was performed by using Surecall as described in the main manuscript text. A threshold for read depth was set of at least 50 times (x axes), and a threshold for coverage at 90% (y axes) of the original regions of interest. The two samples that did not match this criteria cluster clearly separate from the rest (12–04-079_22 and 12–04-079_25). Sample 12–04-079_31 was of borderline sufficient quality.

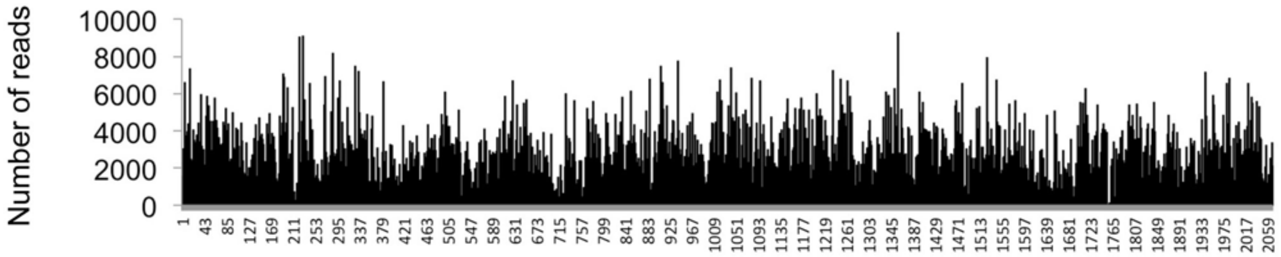


**Supplementary Figure S2: Average coverage of the captured regions.** Average coverage of the 2069 captured regions covering the 154 genes. The samples with low quality (12–04-079_22 and 25) are excluded. Mean reads (plus maximum and minimum) per each region in each sample are given in Supplemental Table S2, which gives also the correspondence between region number (on the x axes) and captured gene exon (with chromosomal coordinates).

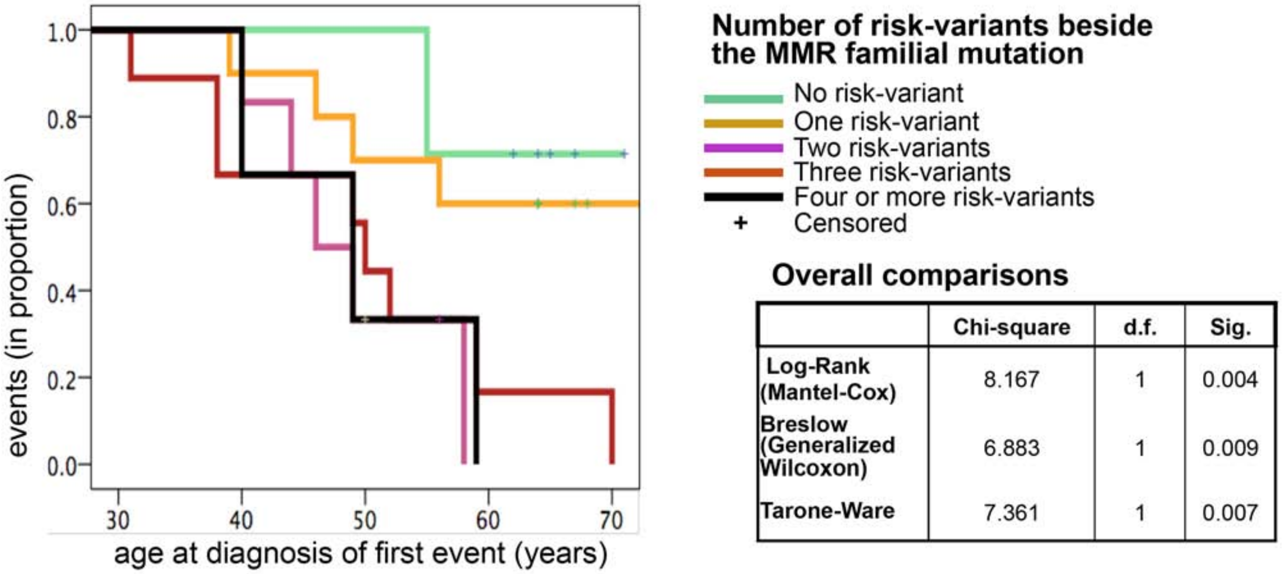


**Overall comparisons**

| | Chi-square | d.f. | Sig. |
|---|---|---|---|
| Log-Rank (Mantel-Cox) | 8.167 | 1 | 0.004 |
| Breslow (Generalized Wilcoxon) | 6.883 | 1 | 0.009 |
| Tarone-Ware | 7.361 | 1 | 0.007 |

| No. risk-variants | lifespan* | total | Any poor-phenotype event | | EC before 50 years | | Second tumour | |
|---|---|---|---|---|---|---|---|---|
| | *p = ns* | n | n | % | n | % | n | % |
| ***0*** | 66 ± *5* | 7 | 2 | 28.6 | 0 | 0.0 | 2 | 28.6 |
| ***1*** | 65 ± *7* | 10 | 4 | 40.0 | 3 | 30.0 | 2 | 20.0 |
| ***2*** | 65 ± *7* | 6 | 5 | 83.3 | 4 | 66.6 | 3 | 50.0 |
| ***3*** | 64 ± *12* | 9 | 8 | 88.9 | 4 | 44.4 | 5 | 55.5 |
| ***4-5*** | 64 ± *23* | 3 | 3 | 100.0 | 2 | 66.6 | 1 | 33.3 |
| ***overall*** | 65 ± *11* | 35 | 22 | 62.9 | 13 | 37.1 | 13 | 37.1 |
| ***Log-Rank*** | | | *0.004* | | *0.02* | | *0.08* | |

**Supplementary Figure S3: Kaplan-Meier analyses of any poor clinical phenotype event: diagnosis of endometrial cancer below 49 years and/or diagnosis of a second cancer.** Kaplan-Meier analysis of the occurrence of any event classified as poor-clinical-phenotype (either diagnosis of endometrial cancer at 49 years or earlier, or the diagnosis of a second tumor other than the endometrial one) among subjects carrying no risk-variants, one, two, three or more than four risk-variants beside the MMR gene familial mutation. Overall comparisons: statistic computed with SPSS; d.f. = degrees of freedom; Sig = value for statistical significance. The table at the bottom shows the overall distributions of the events among the difference categories. * lifespan was extrapolated at 2013. Log-Rank (Mantel-Cox) for statistical significance is shown for the occurrence of any poor phenotype event (shown also as Kaplan-Meier graph, top left), and for the early diagnosis of endometrial cancer or the diagnosis of a second tumor separately. Data in the Kaplan-Meier graph (top left) show that subjects carrying no or one risk-variant beside the MMR mutation cluster separately from subjects carrying two or more risk-variants. In the main text, analyses were performed by categorizing subjects in those carrying no or one risk-variants and those carrying more than two risk-variants beside the MMR mutation.

| FAMILY | NUMBER METC 12-04-079 | TUMOR PHENOTYPE | AGE IN 2013 | Familial MMR mutation | Additional MMR variant | total | Risk-variants outside the MMR genes | | |
|---|---|---|---|---|---|---|---|---|---|
| A | 2 | N | 73 | MSH6 | none | 1 | NCOR2-S1964C | | |
| A | 3 | poor (M) | 71 | MSH6 | none | 3 | NCOR2 S1964C | MGMT P233L | DICER1 A872T[16] |
| E | 19 | N | 62 | MSH2 | rs1800937 | 0 | | | |
| E | 17 | poor (Y) | 58 | MSH2 | none | 2 | PPARGC1A G735V | NOTCH1 P2448S | |
| F | 28 | N | 67 | MSH6 | none | 1 | FANCM L57F[1] | | |
| F | 26 | poor (Y) | 56 | MSH6 | none | 3 | FANCM L57F[1] | EPHB2 C621R[18] | MLL ins3439T |
| B | 6 | N | 56 | MSH6 | none | 3 | CYP1A1 C457fs* | BRCA2 D596H[6] | RET N361K |
| B | 7 | poor (M) | 87 | MSH6 | rs1042821 | 3 | CYP1A1 C457fs* | BRCA2 D596H[6] | RET N361K |
| C | 8 | N | 65 | MSH6 | MSH6-L396V rs2020908 (cat-2) | 0 | | | |
| C | 9 | poor (M) | 63 | MSH6 | MSH6-L396V rs2020908 (cat-2) | 0 | | | |
| G | 29 | poor (Y,M) | 82 | MLH1 | none | 1 | RXFP2 T222P[7] | | |
| G | 18 | poor (Y) | 56 | MLH1 | rs12998837 | 2 | RAD51D D90G[11] | NTRK1 T300A | |
| D | 12 | poor (Y) | 70 | MSH2 | none | 1 | COMT R128H | | |
| D | 11 | poor (Y) | 61 | MSH2 | none | 3 | COMT R128H | ATM L1590F[14] | TSC1 R190C[15] |
| D | 16 | poor (Y,M) | 67 | MSH2 | rs1042821 | 3 | APC Y1624S | BARD1 S761N[19] | ATM L1590F[14] |

**Supplementary Figure S4: Clinical phenotypes and genetic characterization of relatives.** The seven families (15 subjects) for which DNA from relatives was screened. Among the five families in which members have distinct cancer phonotypes (A, E, F, B and C), in three (A, E and F), the member with poor clinical phenotype carries more risk-variants than the one with neutral phenotype. In Family B, both members carry three risk-variants and the woman with neutral phenotype is relatively young compared to her relative, characterize by a poor cancer phenotype. Members if family C (one poor and neutral) carry no risk-variants. The remaining two families comprise women who had all a poor cancer phenotype. All members of these families carries at least one risk-variant. Superscripts refer to dbSNP accession, when applicable: (1) rs142007602; (6) rs56328701; (7) rs121918303; (11) rs180869630;(14) rs35962982; (15) rs118203400; (16) rs149242330; (18) rs149475426; (19) rs142155101.

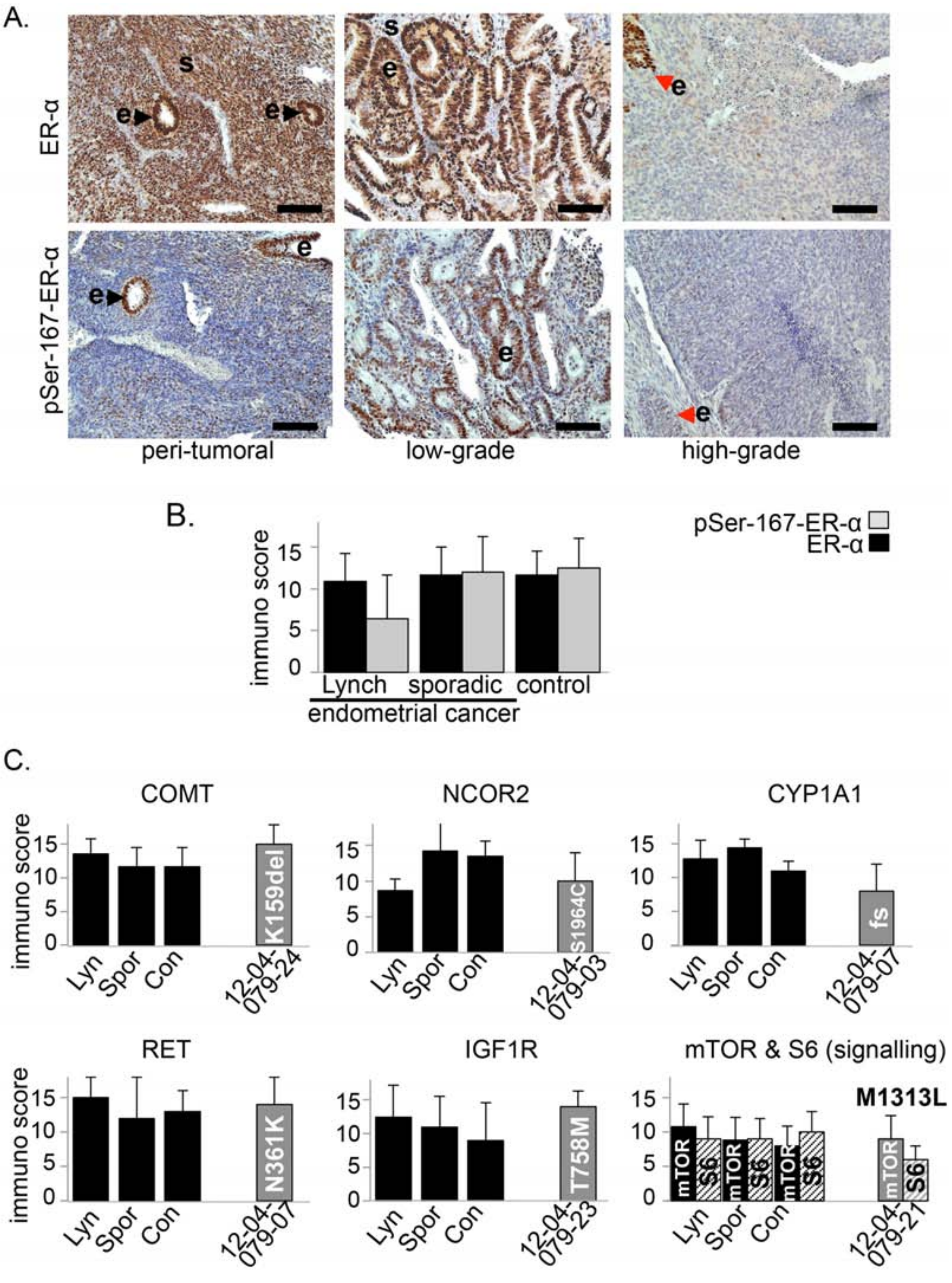


**Supplementary Figure S5: Estrogen signaling and protein expression in tumor specimens. A.** Representative immunohistochemical images showing expression of the estrogen receptor (ER-α) and the phosphorylated form at Ser-167 in normal endometrium adjacent to tumor tissues (peri-tumoral), low-grade and high-grade Lynch syndrome endometrial cancer. 's' marks the stromal component, 'e' the epithelial glandular cells. Stroma is abundant in post-menopausal endometrium and decreases in cancer. In high-grade lesions the cytological differentiation between stromal and epithelial cells is less evident. Red-arrowheads on the images at the right indicate one area of low-grade cancer with clearly well-differentiated epithelial cells. Bar = 100 µm. **B.** Quantification of ER-α (black) and phospho-Ser-167-ER-α (grey) in Lynch syndrome endometrial cancer ($n = 8$), sporadic endometrial cancer ($n = 7$) and post-menopausal controls ($n = 5$). Immuno-scores range: 0–15. Mean values ± standard deviations (SD) are shown. **C.** Quantification (range 0–15) of the immunohistochemical expression of COMT, NCOR2, CYP1A1, RET, IGF1R, mTOR. Expression levels are shown for the group of Lynch syndrome tumors, the group of sporadic tumors, the controls (black bars) and the specimen bearing the indicated risk-variant (grey bars). Black bars are the mean values ± SD of the different subjects (Lynch syndrome specimens without the variant, $n = 7$; sporadic cancers, $n = 7$; controls, $n = 5$). Grey bars are the mean values ± SD of four independent areas of the tumor from the indicated subject bearing the risk-variant.

A

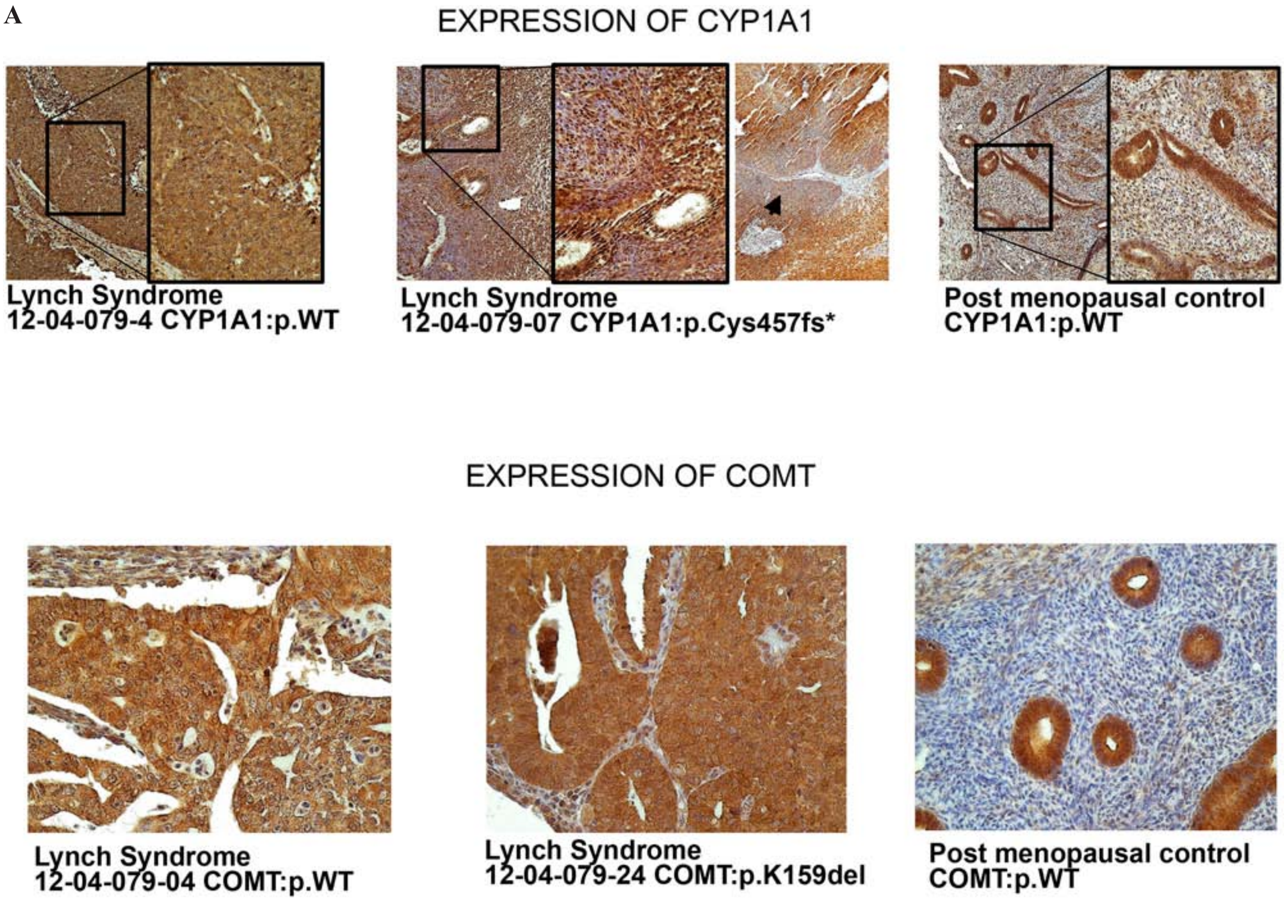


**Supplementary Figure S6: Analysis of tumor material by immunohistochemistry.** Immunohistochemical stainings for CYP1A1, COMT, mTOR (and downstream signal protein S6) and NCOR2. Overall, immunohistochemical analysis (including those for RET and IGF1R in Figure 2 and Supplemental figure 5) shows high expression levels of these proteins (and the downstream signaling activation in case of mTOR) in the endometrium and in endometrial cancer, proofing that they play a role in endometrial physiology. **A.** *(top series)*. Immunohistochemical images of a tumor and a normal endometrium specimens bearing the wild type CYP1A1 or the tumor with risk-variant CYP1A1:p.Cys457fr*, showing the expression of the protein CYP1A1. *(bottom series)*. Immunohistochemical images of a tumor and a normal endometrium specimens bearing the wild type COMT or the risk-variant COMT:p.Lys159del, showing the expression of the protein COMT.

(*Continued*)

**B**

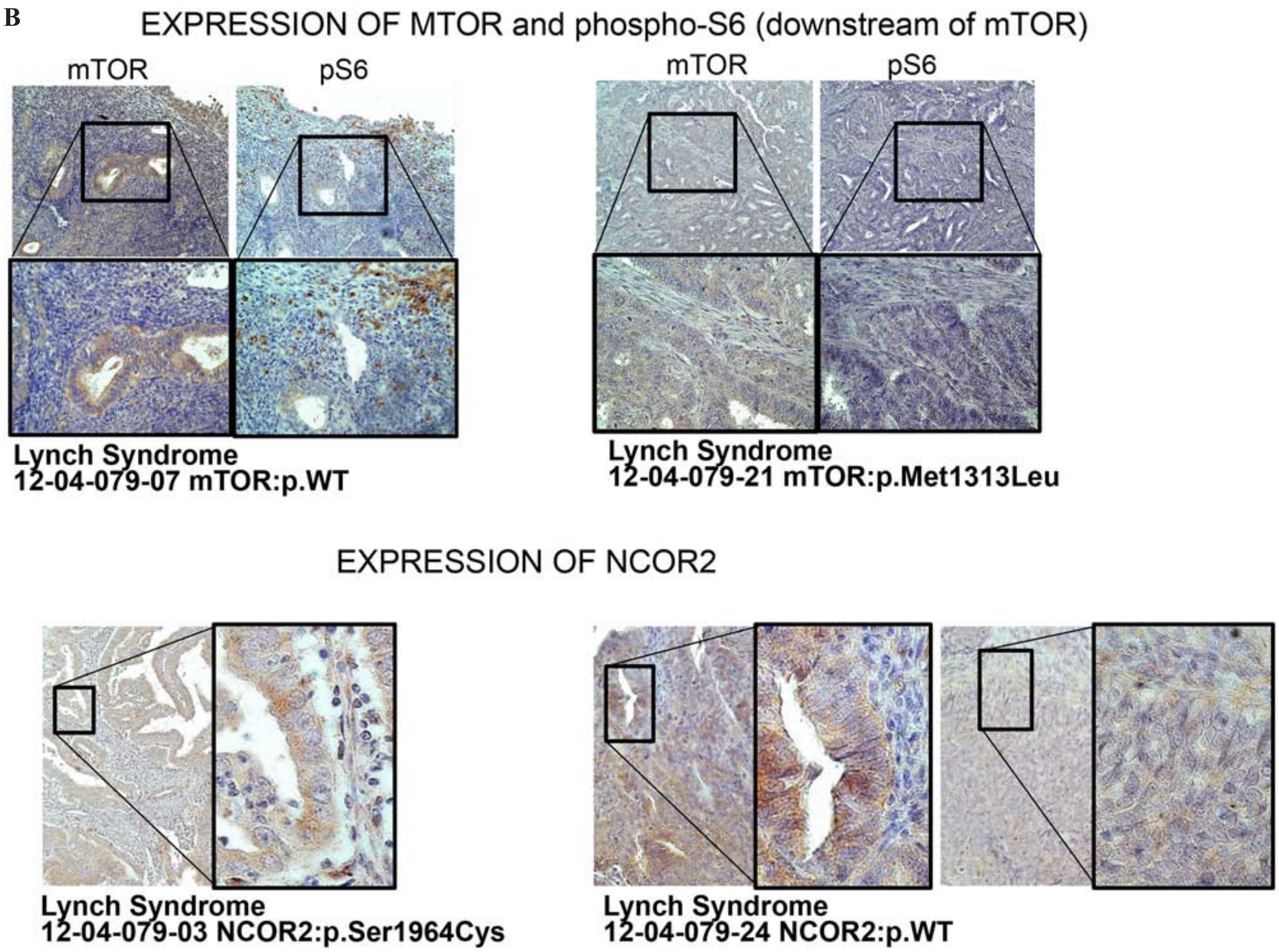


**Supplementary Figure S6: (*Continued*) B.** *(top series)*. Immunohistochemical images of tumors bearing the wild type mTOR or the risk-variant mTOR:p.Met1313Leu, showing the expression of the protein mTOR, phosphorylated S6 (a direct downstream mTOR target that is phosphorylated upon mTOR activation). *(bottom series)*. Immunohistochemical images of tumors bearing the wild type NCOR2 or the risk-variant NCOR2:p.Ser1964Cys and showing the NCOR2 expression. *Immunohistochemistry protocol*: sections of formalin-fixed-paraffin-embedded (FFPE) material were stained using standard protocols (Cornel et al, J Clin Endocrinol Metab. 2012;97:E591–601) and antibody manufacturer's instructions. Tris-EDTA buffer was used to retrieve the antigen for staining. The following antibodies were used to stain for the respective proteins: CYP1A1 (1:1000; polyclonal; Sigma-Aldrich, St. Louis, USA), NCOR2 (SMRT, 1:100; polyclonal; Santa Cruz Biotechnology, Inc., Dallas, USA), COMT (1:100 polyclonal ; Sigma-Aldrich, St. Louis, USA), Phospho-ERα ser167 (1:50; monoclonal; Cell Signaling Technology, Inc., Danvers, USA), Phospho-S6 Ribosomal protein-ser235/236 (1:100; monoclonal; Cell Signaling Technology, Inc., Danvers, USA), mTOR (1:100; monoclonal; Cell Signaling Technology, Inc., Danvers, USA). Chemate Envision and 3,3-diaminobenzidine (DAB) solution (Dako, Glostrup, Denmark) were used to visualize antibody binding.
